# *CASP8* promoter polymorphism, mRNA expression and risk of prostate cancer among Chinese men^☆^

**DOI:** 10.1016/S1674-8301(11)60016-X

**Published:** 2011-03

**Authors:** Guangbo Fu, Jialin Tang, Meilin Wang, Chao Qin, Fu Yan, Qi Ding, Changjun Yin, Xinru Wang, Zhengdong Zhang

**Affiliations:** a Key Laboratory of Reproductive Medicine, School of Public Health, Institute of Toxicology, Nanjing Medical University, Nanjing, Jiangsu 210029, China; b Department of Urology, Huai-An First Affiliated Hospital, Nanjing Medical University, Huai-An, Jiangsu 211000, China; c Jiangsu Key Laboratory of Cancer Biomarkers, Prevention & Treatment, Cancer Center, Nanjing Medical University, Nanjing, Jiangsu 210029, China; d Department of Molecular & Genetic Toxicology, School of Public Health, Cancer Center, Nanjing Medical University, Nanjing, Jiangsu 210029, China; e Department of Urology, the First Affiliated Hospital of Nanjing Medical University, Nanjing, Jiangsu 210029, China

**Keywords:** caspase-8, promoter polymorphism, genetic variation, prostate cancer

## Abstract

Caspase-8 (CASP8) plays a key role in apoptosis. We examined by genotyping whether the -652 six-nucleotide insertion-deletion (6N ins/del) polymorphism in the *CASP8* promoter region was associated with prostate cancer risk in a hospital-based case-control study of 406 Chinese prostate cancer patients and 408 age-matched cancer-free controls. Additionally, 23 prostate cancer tissues were analyzed for *CASP8* mRNA expression. We found a significantly decreased prostate cancer risk for the 6N ins/del genotype [adjusted odds ratio (*OR*)=0.68; 95% confidence interval (*CI*)=0.51-0.92] and del/del genotype (*OR*=0.34; 95% *CI*=0.19-0.63) compared with the ins/ins genotype. The 6N del allele was associated dose-dependently with decreased prostate cancer risk (*P*_trend_ = 0.001). RT-PCR showed that individuals with the 6N del allele had lower *CASP8* mRNA levels than those with the ins/ins genotype (*P* = 0.024). These findings suggested that the *CASP8*-652 6N ins/del polymorphism may affect the susceptibility to prostate cancer and reduce prostate cancer risk among Chinese men.

## INTRODUCTION

Prostate cancer is the most common diagnosed malignant cancer and the second leading cause of cancer deaths in men in the United States, with an estimated 192,280 new cases and 27,360 deaths in 2009[1]. The well-known risk factors for prostate cancer are age, family history of prostate cancer and ethnicity[2]. Ethnic differences in the incidence of prostate cancer have been well established, with the highest rates in Western countries and lowest in Asia countries[3],[4]. Although the prostate cancer rate is lower in China than that in Western countries, the incidence of prostate cancer has increased rapidly among Chinese men over the recent years[5]. As shown in previous studies, both genetic and environmental factors are involved in the development of prostate cancer[6]–[8].

Apoptosis is an essential protective mechanism against excessive cellular proliferation and malignancy[9]. Cells are persistently in a balance between proliferation and apoptosis, and dysfunction of this balance may increase the risk of cancer[10]. Studies have reported that caspases are at the crossroads of immune cell life and death, and their aberrant expressions or activities are associated with many pathological conditions, including cancer[11]. Caspase-8 (CASP8) is a crucial member of the caspase family in the apoptotic process[12]. Therefore, genetic variants in *CASP8* may disturb the function of CASP8 and then affect the execution of apoptosis, thus contributing to either the development or progression of human cancers.

Accumulating studies have reported that two common single nucleotide polymorphisms (SNPs) in the *CASP8* promoter gene, -652 6N ins/del (rs3834129) and D302H (rs1045485), were associated with risk of cancers[13]–[21]. Studies have revealed that the polymorphism in the *CASP8* coding region, D302H, was associated with reduced breast cancer risk[13],[15],[20]. Study showed that histidine variant of *CASP8* D302H is a protective allele for aggressive prostate cancer[21]. However, the *CASP8* D302H variant showed low-penetrance in Asians (minor allele frequency, MAF = 0% in Asians, MAF = 12.5% in Europeans based on the HapMap and Environmental Genome Project database), and we could not analyze the association with prostate cancer risk in this study. Sun *et al*[18]. identified that the -652 6N ins/del polymorphism in the *CASP8* promoter region was associated with several types of cancer in Chinese population, and the 6N del variant decreased mRNA expression or activity by destroying a binding site for stimulatory protein (Sp1) and protected against cancers. However, Haiman *et al.*[22] showed that the *CASP8* -652 6N ins/del polymorphism was not associated with risk of cancers including prostate cancer in a multiethnic cohort study. To explore the potential association between the *CASP8* -652 6N ins/del polymorphism and prostate cancer risk in a Chinese population, we performed a hospital-based case-control study with 406 prostate cancer cases and 408 cancer-free controls. Meanwhile, we evaluated the relationship between *CASP8* mRNA expression and the -652 6N ins/del genotypes in prostate cancer tissue.

## MATERIALS AND METHODS

### Study population

This study consisted of 406 prostate cancer cases and 408 cancer-free controls. All subjects were unrelated southern Han Chinese. Cases were diagnosed with prostate cancer by needle biopsy and recruited from the First Affiliated Hospital of Nanjing Medical University between September 2003 and July 2009. The control subjects were recruited from healthy people who were seeking health care in the outpatient departments at the hospital. We used a questionnaire to obtain detailed information about demographic data and risk factors (e.g., age, race, smoking status, alcohol use, and family history of cancer) through face-to-face interviews, we frequency-matched the controls to the cases by age (±5 years).

Before recruitment, informed consent was obtained from each of the eligible subjects. Subjects who smoked one cigarette daily for more than one year were defined as ever smokers and the others as never smokers. Pack-years of smoking [(cigarettes per day / 20)×years smoked] were calculated to indicate the cumulative smoking dose. Individuals that consumed three or more alcohol drinks per week for at least six months were considered as ever drinkers, and the others were defined as never drinkers. Family history of cancer was defined as any cancer in first-degree relatives (parents, siblings, or children). Clinical stage was determined by pathologic results, pelvic computed tomography (CT), magnetic resonance image (MRI), and radio-nucleotide bone scans. According to the international tumor-node-metastasis (TNM) staging system for prostate cancer, the clinical stage was classified into localized and advanced stage (localized: T_1-2_N_0_M_0_; advanced: T_3-4_N_x_M_x_ or T_x_N_1_M_x_ or T_x_N_x_M_1_). Pathologic grade was recorded based on the Gleason score. The response rate for recruitment was above 95% and 85% of the cases and controls, respectively. Each subject donated 5 mL of peripheral blood for genomic DNA extraction. Twenty-three prostate cancer tissues were collected from patients who were diagnosed with prostate cancer and underwent prostatectomy at the First Affiliated Hospital of Nanjing Medical University. All tissues were stored in liquid nitrogen. The research protocol was approved by the institutional review board of Nanjing Medical University.

### Genotyping

According to the NCBI public SNP database, one common SNP (MAF ≥ 5% in Chinese population) rs3834129 six nucleotide insertion/deletion (6N ins/del) polymorphism was selected. The *CASP8* -652 6N ins/del polymorphism was genotyped using the polymerase chain reaction-restriction fragment length polymorphism (PCR-RFLP) method. The primers, lengths, and restriction enzyme were described previously[18]. Genotyping was done without knowledge of the case and control status. Polymorphism analysis was performed by two persons independently. A 10% random sample were tested for conformation, and the concordance was 100%.

### Analysis of *CASP8* mRNA expression

To further explore the correlation between the *CASP8* mRNA expression and -652 6N ins/del genotypes *in vivo*, we extracted total RNA from 23 prostate cancer tissues with different genotypes using Trizol Reagent (Invitrogen, Carlsbad, CA, USA) according to the manufacturer's protocol. The total RNA was measured by reverse transcription-PCR (RT-PCR) and quantitative real-time PCR (qRT-PCR) (ABI Prism^®^ 7900HT quantitative real-time PCR instrument). *GAPDH* was used as an internal quantitative control. The detailed methods for RT-PCR and qRT-PCR were described previously[23]. The primers used for amplification of *CASP8* mRNA were 5′-CATCCAGTCACTTTGCCAGA 3′ (sense) and 5′-GCATCTGTTTCCCCATGTTT 3′ (antisense), and the primers for *GAPDH* were 5′'-GAAATCCCATCACCATCTTCCAGG- 3′ (sense) and 5′-GAGCCCCAGCCTTCTCCATG - 3′ (antisense). Fold changes were normalized by the levels of *GAPDH* expression, and each assay was done in triplicate.

### Statistical analysis

Hardy-Weinberg equilibrium (HWE) of the genotype distributions among controls was tested by a goodness-of-fit χ^2^ test. Chi-square (χ^2^) test was used to analyze the differences in variable frequency distributions, the known risk factors, *CASP8* -652 6N ins/del genotypes and allele frequencies between the cases and controls. Unconditional univariate and multivariate logistic regression analysis were done to obtain the crude and adjusted odds ratios (ORs) and their 95% confidence intervals (CIs). The multivariate analysis was adjusted for age, smoking status and alcohol use. For mRNA quantitative analysis, unpaired *t*-test was performed to analyze the *CASP8* mRNA expression levels (2^−△△CT^). *P* < 0.05 was used as the criterion of statistical significance and all tests were two-sided and analyzed by using the SAS software (Version 9.1; SAS Institute, Inc., Cary, NC, USA).

## RESULTS

### Characteristics of the study subjects

The frequency distributions of selected variables between the cases and controls are shown in ***Table 1***. There was no difference in the distribution of age between the cases and controls (*P* = 0.205). However, there were more smokers (59.8% *vs* 49.7%, *P* = 0.001) and drinkers (30.0% *vs* 23.3%, *P* = 0.029) among cases than controls. Specifically, the frequency of family members with cancer from the case group was higher than the control group (28.3% *vs* 16.2%, *P* < 0.001). Among the cases, 218 (53.7%) patients were in localized stage and 188 (46.3%) were in advanced stage. The distributions of Gleason score in < 7, = 7, and > 7 subgroups among cases were 173 (42.6%), 106 (26.1%) and 127 (31.3%), respectively. Furthermore, the prostate antigen (PSA) levels were separated into two groups: ≤ 20 ng/mL (170, 41.9%) and > 20 ng/mL (236, 58.1%).

**Table 1:** Distributions of selected variables between prostate cancer cases and cancer-free controls

Variables	Cases (*n* = 406)	Controls (*n* = 408)	*P^a^*
	*n*(%)	*n*(%)	
Age (years) (mean ± SD)	69.4 ± 6.6	69.3 ± 5.5	0.916
< 70	183 (45.1)	202 (49.5)	0.205
≥ 70	223 (54.9)	206 (50.5)	
Smoking status			0.001
Never	163 (40.2)	205 (50.3)	
Ever	243 (59.8)	203 (49.7)	
Pack-years ≤ 28	110(27.1)	115(28.2)	
Pack-years > 28	133 (32.7)	88 (21.5)	
Alcohol drinking			0.029
Never	284 (70.0)	313 (76.7)	
Ever	122 (30.0)	95 (23.3)	
Family history of cancers			< 0.001
No	291 (71.7)	342 (83.8)	
Yes	115 (28.3)	66 (16.2)	
Clinical stage			
Localized	218 (53.7)		
Advanced	188 (46.3)		
Gleason (score)			
< 7	173 (42.6)		
= 7	106 (26.1)		
> 7	127 (31.3)		
PSA (ng/mL)			
≤ 20	170 (41.9)		
>20	236 (58.1)		

^a^ *T*-test for age distributions between the cases and controls; two-sided χ^2^ test for the other selected variables between the cases and controls. PSA: prostate specific antigen.

### Association between *CASP8* -652 6N ins/del polymorphism and prostate cancer risk

The genotype and allele frequencies of the *CASP8* -652 6N ins/del polymorphism in the cases and controls and their associations with prostate cancer risk are shown in ***Table 2***. The observed genotype frequency among the control subjects was in agreement with the HWE (χ^2^ = 1.01, *P* = 0.315). The frequencies of the 6N ins/ins, ins/del, and del/del genotypes were 63.3%, 32.5%, and 4.2%, respectively, among the cases, and 51.7%, 39.0%, and 9.3%, respectively, among the controls (*P* = 0.001). The del allele frequencies were 0.20 among the cases and 0.29 among the controls (*P* < 0.001). As shown in ***Table 2***, when we used the 6N ins/ins genotype as the reference, we found that both 6N ins/del and del/del genotypes were associated with a statistically significantly decreased risk of prostate cancer (adjusted *OR* = 0.68, 95% *CI* = 0.51-0.92 for 6N ins/del and adjusted *OR* = 0.34, 95% *CI* = 0.19-0.63 for 6N del/del). We also found that the risk of prostate cancer associated with the del allele was decreased in a dose-response manner (*P*_trend_ = 0.001). In the stratification analysis, as shown in ***Table 3***, we found that the decreased risk of prostate cancer was pronounced among subgroups of age≥ 70 (*CI* = 0.47, 95%CI 0.31-0.69), smokers and non-drinkers (*OR* = 0.63, 95%*CI* = 0.46-0.88). As shown in ***Table 4***, when the cases were stratified by clinicopathological characteristics (i.e., clinical stage, Gleason score and PSA level) and compared with the controls, we found that the associations were all statistically significant. However, when we used the localized clinical stage, Gleason < 7 and PSA ≤ 20 as control, we found no trends (data not shown).

**Table 2:** Genotype and allele frequencies of the *CASP8* -652 6N ins/del polymorphism among cases and controls and their associations with risk of prostate cancer

*CASP8*	Cases (*n* = 406)	Controls (*n* = 408)	Adjusted *OR* (95% *CI*)^a^	*P*^b^
	*n* (%)	*n* (%)		
Genotypes				0.001
ins/ins	257 (63.3)	211 (51.7)	1.00	
ins/del	132 (32.5)	159 (39.0)	0.68 (0.51-0.92)	0.011
del/del	17 (4.2)	38 (9.3)	0.34 (0.19-0.63)	0.001
del allele	0.20	0.29		< 0.001
*P*_trend_				0.001

^a^ Adjusted for age, smoking and drinking status; ^b^ Two-sided χ^2^ test. OR: odds ratio.

**Table 3:** Stratification analysis between the *CASP8* -652 6N ins/del polymorphism and risk of prostate cancer in cases and controls

Variables	*n*	Genotypes				Adjusted *OR* (95% *CI*)^a^	*P^b^*
	Cases/Controls	ins/ins [n (%)]		ins/del±del/del [n (%)]			
		Case	Control	Case	Control		
Total	406/408	257 (63.3)	211 (51.7)	149 (36.7)	197 (48.3)	0.62 (0.47-0.82)	0.001
Age							
<70	183/202	107 (58.5)	110 (54.5)	76 (41.5)	92 (45.5)	0.86 (0.57-1.30)	0.428
≥ 70	223/206	150 (67.3)	101 (49.0)	73 (32.7)	105 (51.0)	0.47 (0.31-0.69)	0.001
Pack-years							
0	163/205	102 (62.6)	114 (55.6)	61 (37.4)	91 (44.4)	0.75 (0.50-1.15)	0.178
0-28	110/115	68 (61.8)	52 (45.2)	42 (38.2)	63 (54.8)	0.51 (0.30-0.87)	0.013
>28	133/88	87 (65.4)	45(51.1)	46 (34.6)	43 (48.9)	0.56 (0.32-0.97)	0.034
Drinking status							
No	284/313	180 (63.4)	163 (50.1)	104 (36.6)	150 (47.9)	0.63 (0.46-0.88)	0.005
Yes	122/95	77 (63.1)	48 (50.5)	45 (36.9)	47 (49.5)	0.61 (0.35-1.05)	0.063

^a^ Two-sided χ^2^ test; ^b^ Adjusted for age, smoking and drinking status. CI: confidence interval; OR: odds ratio.

**Table 4:** Associations between the *CASP8* -652 6N ins/del polymorphism and clinicopathological characteristics of prostate cancer variables

Variables	Genotype		Adjusted *OR* (95% *CI*)^a^	*P^b^*
	ins/ins [n (%)]	ins/del +del/del [n (%)]		
Controls (*n* = 408)	211 (51.7)	197 (48.3)	1.00	
Cases (*n* = 406)	257 (63.3)	149 (36.7)	0.62 (0.47-0.82)	0.001
Clinical stage				
Localized	141 (64.7)	77 (35.3)	0.62 (0.41-0.93)	0.002
Advanced	116(61.7)	72 (38.3)	0.62 (0.45-0.85)	0.023
Gleason (score)				
<7	106 (61.3)	67 (38.7)	0.67 (0.47-0.97)	0.034
= 7	71 (67.0)	35 (33.0)	0.50 (0.31-0.79)	0.005
>7	80 (63.0)	47 (37.0)	0.64 (0.43-0.97)	0.026
PSA (ng/mL)				
≤20	106 (62.4)	64 (37.6)	0.63 (0.44-0.91)	0.017
>20	151 (64.0)	85 (36.0)	0.61 (0.44-0.84)	0.003

^a^ Two-sided χ^2^ test; ^b^ Adjusted for age, smoking and drinking status. CI: confidence interval; OR: odds ratio; PSA: prostate antigen.

### Association of the *CASP8* -652 6N ins/del polymorphism with expression levels of *CASP8* mRNA

**Fig. 1:**
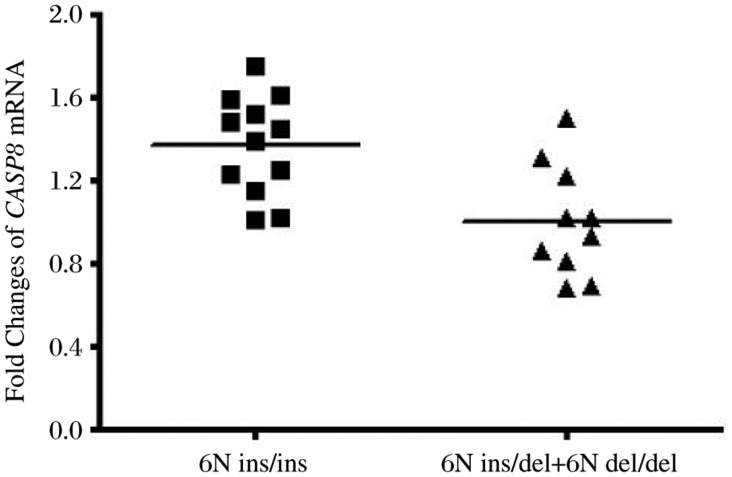
Association between the *CASP8*-652 6N ins/del polymorphism and mRNA levels. *CASP8* mRNA transcript in prostate cancer tissues was assessed by quantitative real-time PCR. The frequency distributions of the 6N ins/ins, 6N ins/del, and 6N del/del genotypes were 12, 10, and 1, respectively. The fold change was normalized against *GAPDH*. *, *P* < 0.05 compared with 6N ins/ins genotype.

In the present study, 23 prostate cancer tissues were obtained from the untreated prostate cancer patients with different genotypes of the 6Nins/del polymorphism. We found that the frequency distribution of the 6N ins/ins, ins/del, del/del genotypes was 12, 10 and 1, respectively. Because only one sample with the del/del genotype was obtained, we added it to the samples with ins/del genotype for analysis. The associations between the two groups and *CASP8* mRNA expression were performed by using qRT-PCR. As shown in ***Fig. 1***, the level of *CASP8* mRNA was lower in individuals with the del allele than in those with the 6N ins/ins genotype, and this difference was statistically significant (6N ins/ins *vs* 6N ins/del + del/del: 1.317±0.25 *vs* 1.000±0.28, *P* = 0.024, in arbitrary units).

## DISCUSSION

In this study, we identified that the *CASP8* -652 6N ins/del polymorphism was associated with reduced risk of prostate cancer. Specifically, the risk of prostate cancer associated with the variant genotypes with the 6N del allele was decreased in a dose-dependent manner. In the stratification analysis, we found that the decreased risk of prostate cancer was more pronounced among the subgroups of age ≥70 years, smokers and non-drinkers. Furthermore, we observed that the 6N ins/del + del/del genotypes were associated with lower *CASP8* mRNA expression levels, similar to the published results that the 6N del variant could reduce *CASP8* expression or activity reported by Sun *et al*.[18]. This is the first study of the association between *CASP8* -652 6N ins/del polymorphism and prostate cancer risk in Chinese population.

Apoptosis is an important and complicated process. Caspases are a conserved family of cysteine proteases that are responsible for cellular apoptosis. CASP8 is an essential regulator of apoptosis[24]. The two common polymorphisms in the *CASP8* gene, -652 6N ins/del and D302H, have been well studied. However, Frank *et al*.[25] reported that the *CASP8* -652 6N ins/del polymorphism was not associated with breast cancer in Europeans. Additionally, Pittman *et al*.[26] failed to replicate this association with colorectal cancer risk. In a multiethnic cohort study, Haiman *et al*.[22] revealed that a promoter polymorphism in the *CASP8* gene was not associated with risk of breast cancer, colorectal cancer and prostate cancer. In the present study, we found that the *CASP8* -652 6N ins/del polymorphism was associated with reduced risk of prostate cancer in a Chinese population. Meanwhile, we obtained the corresponding association between *CASP8* mRNA expression and 6N ins/del polymorphism. The -652 6N del variant reduced *CASP8* mRNA expression by removing a binding site for the transcriptional activator Sp1 in lymphocytes, and T lymphocytes with the del allele had lower *CASP8* activity[18]. Explanation of the discrepancy may be the differences in genetic backgrounds, study populations and study samples. The differences may reflect the fact that the association between the *CASP8* -652 6N ins/del polymorphism and the decreased cancer risk is more dominant in Chinese populations. These may explain the low incidence of prostate cancer in China from some aspects.

To our best knowledge, age is an important risk factor of prostate cancer, and the incidence of prostate cancer has shown an increasing trend in elderly men[2],[27]. Tobacco smoking is shown to be significantly associated with higher lung cancer risk and moderately increased all cancer risk[28]. In a word, increasing age and tobacco smoking are risk factors for health. At the same time, moderate alcohol drinking is good for health[29]. However, we found that the protective effect of the 6N del allele was more pronounced in subgroups of age ≥ 70 years, smokers, and non-drinkers. The probable interpretation of this discrepancy may be that when individuals are exposed to relative dangerous environmental factors, the protective effect of the 6N del allele becomes more dominant to protect individuals. Meanwhile, stratification analysis of clinicopathological characteristics showed that the associations were statistically significant when each subgroup was compared with controls, and the association between cases and controls was statistically significant (adjusted OR = 0.62, 95% CI = 0.47-0.82). However, there were no statistically significant trends when we used localized clinical stage, Gleason < 7 and PSA ≤20 ng/mL as control (data not shown).

Several limitations in this study should be addressed. Firstly, our study was a hospital-based case-control study, which could not evacuate the possibility of selection bias of subjects. However, the genotype distributions in our research are consistent with HWE (χ^2^ = 1.01, *P* = 0.315) and the results are similar to the published data in Chinese population[18],[19]. For instance, the frequencies of the ins/ins, ins/del, del/del genotypes among our controls were 51.7%, 39.0% and 9.3%, respectively, compared to 55.7%, 37.5% and 6.8% in the study by Wang *et al*.[19] and 56.3%, 37.2% and 6.5% in the study by Sun *et al*.[18]. Secondly, our study sample size was relatively small, which may limit the statistical power. Larger sample size studies are needed to validate these findings. Thirdly, only one sample with the del/del genotype was obtained among the 23 prostate cancer tissues; thus, we added it to the samples with the ins/del genotype for analysis. More samples of prostate cancer tissues are needed for more in-depth studies.

In conclusion, this study identified that the *CASP8* -652 6N ins/del polymorphism was associated with reduced risk of prostate cancer in a Chinese population.
